# Systematic analysis of NLMP suggests nuclear localization of RTK/MET kinases resemble cancer cell clearance

**DOI:** 10.1186/s13046-018-1004-z

**Published:** 2019-01-30

**Authors:** Yingqiu Xie, Ayan A. Nurkesh, Nazgul Ibragimova, Zhuldyz Zhanzak, Aizhan Meyerbekova, Zhanna Alexeyeva, Aiya Yesbolatova, Madina Satayeva, Aidana Mustafa, Limara Manarbek, Aisulu Maipas, Akerke Altaikyzy, Zhibek Keneskhanova, Burkitkan Akbay, Zhenbang Chen

**Affiliations:** 1 Department of Biology, School of Science and Technology, Nazarbayev University, Qabanbay Batyr Ave 53, Astana, 010000 Kazakhstan; 2 Department of Biochemistry and Cancer Biology, Meharry Medical College, Nashville, TN 37208 USA

**Keywords:** Nuclear localized membrane protein (NLMP), MET, Cell death, Drug resistance, Cancer evolution

## Abstract

**Background:**

Some membrane proteins can translocate into the nucleus, defined as nuclear localized membrane proteins (NLMPs), including receptor tyrosine kinases (RTKs). We previously showed that nuclear MET (nMET), a member of RTKs, mediates cancer stem-like cells self-renewal to promote cancer recurrence. However, it is unknown that nMET or mMET, which is the ancestor in the evolution of cancer cell survival and clearance. Here, we aim to study the NLMP functions in cell death, differentiation and survival.

**Method:**

We applied the systematic reanalysis of functional NLMP and clinical investigations of nMET from databases. In addition, we used soft agar assay, immunoblotting, flow cytometry, and immunofluorescence confocal microscopy for examinations of nMET functions including stem-like cell formation, cell signaling, cell cycle regulation, and co-localization with regulators of cell signaling. ShRNA, antibody of recognizing surface membrane MET based treatment were used to downregulate endogenous nMET to uncover its function.

**Results:**

We predicted and demonstrated that nMET and nEGFR are most likely not ancestors. nMET overexpression induces both cell death and survival with drug resistance and stem cell-like characters. Moreover, the paradoxical function of nMET in both cell death and cell survival is explained by the fact that nMET induces stem cell-like cell growth, DNA damage repair, to evade the drug sensitization for survival of single cells while non-stem cell-like nMET expressing single cells may undergo clearance by cell death through cell cycle arrest induced by p21.

**Conclusion:**

Taken together, our data suggest a link between nuclear RTK and cancer cell evolutionary clearance via cell death, and drug resistance for survival through stemness selection. Targeting evolved nuclear RTKs in cancer stem cells would be a novel avenue for precision cancer therapy.

**Electronic supplementary material:**

The online version of this article (10.1186/s13046-018-1004-z) contains supplementary material, which is available to authorized users.

## Introduction

Cellular translocation of proteins is one of the important events of communication between cellular compartments. Most proteins can reach their targets by specific regulation of localization being under the co-translational or post-translational stage [1, 2]. For nuclear localization, several proteins translocate by diffusion-retention mechanism. Other proteins may possess nuclear localization signal (NLS) which is usually recognized by adaptors α/β importins [2] to be imported to nucleus passing nuclear pore. Some membrane proteins may translocate to nucleus and exert various functions such as transcriptional regulation [2, 3]. These proteins may be defined as nuclear localized membrane proteins (NLMPs). One of the big family members of NLMP is receptor tyrosine kinase family (RTK) which can be translocated into nucleus [3, 4].

RTKs are originally identified as transmembrane proteins, which act as receptors and modulate an intracellular signal transduction to initiate pathways of cascade that transfer signal molecules from the membrane to differential compartments [5]. A large number of subfamilies of human RTKs are therapeutic targets in many types of cancers [5]. It has been shown that RTKs can enter the nucleus through nuclear pore and importins [6–8]. Nuclear translocation of RTKs have been shown to be associated with therapeutic resistance, transcriptional regulation and signaling related to DNA replication and DNA damage repair [9, 10]. MET kinase of RTK, which refers to hepatocyte growth factor receptor family member, usually localizes at membrane (mMET) but containing a cytoplasmic tail [11]. MET has also been reported in many cancers to be localized into the nucleus [12, 13]. Nuclear translocation of MET can be through NLS or cleavage [14, 15]. It has been shown that nuclear MET (nMET) can regulate nuclear Ca^2+^ or YAP signaling to stimulate cell proliferation [12, 13] or induce SOX9 and β-catenin to enhance cancer stem-like cells’ self-renewal for cancer recurrence [14]. It has been discovered that total MET induce tumor-initiating, which mediates therapeutic resistance and tumor recurrence [16]. RTKs inhibitors are widely used in conventional therapy. However, in many cancers, RTKs may induce crosstalk-signaling pathways [17] to develop drug resistance [17, 18]. Based on cancer stem cell (CSC) hypothesis, the population of cells expresses high heterogeneity as small part of cells with self-renewal abilities of CSCs. The smaller population of CSCs exists, the higher probability of fast adaptation to microenvironment [17, 18]. Eventually, resembling Darwin’s theory of evolution through natural selection, namely, only cells, which can resist to the stressed microenvironment, will survive [18]. Here using nMET as a case, we tested whether nuclear RTK is essential in cancer evolution through clearance and Darwin’s “Survival of the Fittest” theory via cancer stemness.

## Materials and methods

### Alignment analysis

To find main functions of NMLP proteins, Google Scholar, Nucleotide and Protein databases were screened for transmembrane proteins with NLS. To determine the evolutionary relationship between NLS and TM domains human sequences were used as reference and EGFR, MET alignments were performed as described previously [19]. Multiple sequence alignment with Uniprot was followed by editing, analysis, and further construction of 2 phylogenetic trees (membranous and nuclear MET) using Jalview software [20–23] followed by counting the number of mutations in amino acids of NLS and TM sequences using published data as references [24, 25]. Finally, graphs were obtained to observe the trend in NLS/TM evolution. In brief, MET sequences of NLS are H1068-H1079, HVVIGPSSLIVH [24]; and transmembrane sequences are 933–955 (https://www.uniprot.org/uniprot/P08581) GLIAGVVS ISTALLLLLGFFLWL.

STRING database was used to compare protein-protein interaction maps for both mMET and nMET [26, 27] using whole protein sequence of MET. For nMET interaction map additional partner proteins, such as YAP [28], were inserted into request before running of STRING program, because there is a low number of research papers dedicated to nMET protein. STRING program generated proteins were divided into 3 orders, according to its interaction extent with MET protein.

### Cell lines, cell culture, transfection, and MET knockdown

PC3, MCF7, C4-2B, HEK293, HeLa cells (ATCC) were grown in RPMI 1640 (ThermoFisher Scientific) or DMEM (Invitrogen, USA) with 10% FBS (Invitrogen). For cell transfection with plasmids, Lipofectamine 3000 with Lipofectamine 2000 (ThermoFisher Scientific) was applied. The plasmids pLenti-cytoMetGFP with nuclear MET-GFP genes and pLenti-MetGFP with full length MET-GFP genes were gifts from David Rimm (Addgene plasmid # 37561 and 37560). The plasmids express a truncated form of MET which predominantly localizes to nucleus or full length MET which predominantly localizes to membrane and hardly in cytosol or nucleus [14, 26]. MET knockdown in cells was performed as described previously [14].

### Immunofluorescent staining and microscopy

Cells were fixed for 20 min using 4% formaldehyde solution in 1xPBS followed by washing with PBS and 1 h blocking. Then cells were stained with primary antibodies (15-18 h in 4 °C). Cells were washed with PBS before incubation at room temperature with diluted secondary antibodies (Life Technologies) with dilution buffer containing 0.3% Triton™ X-100 (Sigma-Aldrich) for 1 h. Finally, cells were stained with DAPI and mounted with Fluoromount Aqueous Mounting Medium (Sigma-Aldrich). Images of samples were taken, processed, and analyzed with Carl Zeiss LSM 780 confocal microscope and Zen software (Zeiss).

### Western blotting

Protein samples were collected from cells using NP40 Cell Lysis Buffer (Life Technologies) with Protease Inhibitor (100x, Thermo Scientific) for 30 min. SDS-PAGE gel electrophoresis was performed by running at 100 V in Tris/Gly/SDS running buffer, transferring on 0.45 μm PVDF membrane (Millipore) at 90 V or overnight at 60 V in 1x transfer buffer (Tris/Glycine/Methanol) followed by 1 h incubation in blocking buffer (2% BSA diluted with 1xPBST). Antibodies used are: β-actin (AC-74, Sigma), p21 (Santa Cruz), p53 (Santa Cruz), γH2AX (Cell Signaling), Caspase 3 (Santa Cruz), MET (Cell Signaling), MET (Abcam), PARP (Cell Signaling), Bcl-2 (Sigma), RAD51 (Abcam), SOX2(Cell Signaling), OCT4 (Cell Signaling). After incubation with secondary antibodies and washing with PBST, membranes were analyzed using LI-COR Odyssey imaging system and Image Studio Lite software.

### Cell growth, cell cycle and soft agar assays

Cells were treated with MET antibody (Santa Cruz) on 24-well plates, cultured for additional 3 days with different concentrations of drugs, fixed, and washed 3 times. Crystal violet staining was performed after fixing of cells, followed by 5 times washing with water. For cell cycle assay, cells were transfected by plasmid containing vehicle or nMET (Addgene) as described above and subjected to fixation by 70% ethanol followed by protocol provided by the manufacture using Muse® Cell Cycle Assay Kit (Cat# MCH100106, Merck) with Muse cell analyzer and analysis (Merck).

For growing colonies in soft agar [29] in 6 well plates, cells were resuspended in 0.4% agarose top layer and seeded on 0.6% agarose base layer. The operation was under sterile conditions by mixing medium containing 20% FBS in 6-well plates and cells were grown for 3–4 weeks (37 °C, 5% CO_2_) with further feeding medium with or without membrane MET recognized antibody (Abcam, EP1454Y) for inhibiting mMET treatment. Finally, formed colonies were stained with Crystal Violet, or directly observed and counted under light microscope.

### Clinical data search and analysis

The survival rate of patients was assessed using PubMed database. The following keywords were used as “nuclear translocation/localisation of receptor tyrosine kinase in patients, clinical data”. This allowed to obtain published data to study the relation between the nuclear localized receptor tyrosine kinase and patients’ survival or prognosis [30–47].

## Results

### Systematic analysis suggests the paradoxical function of NLMP: Both cell death and survival

**Fig. 1:**
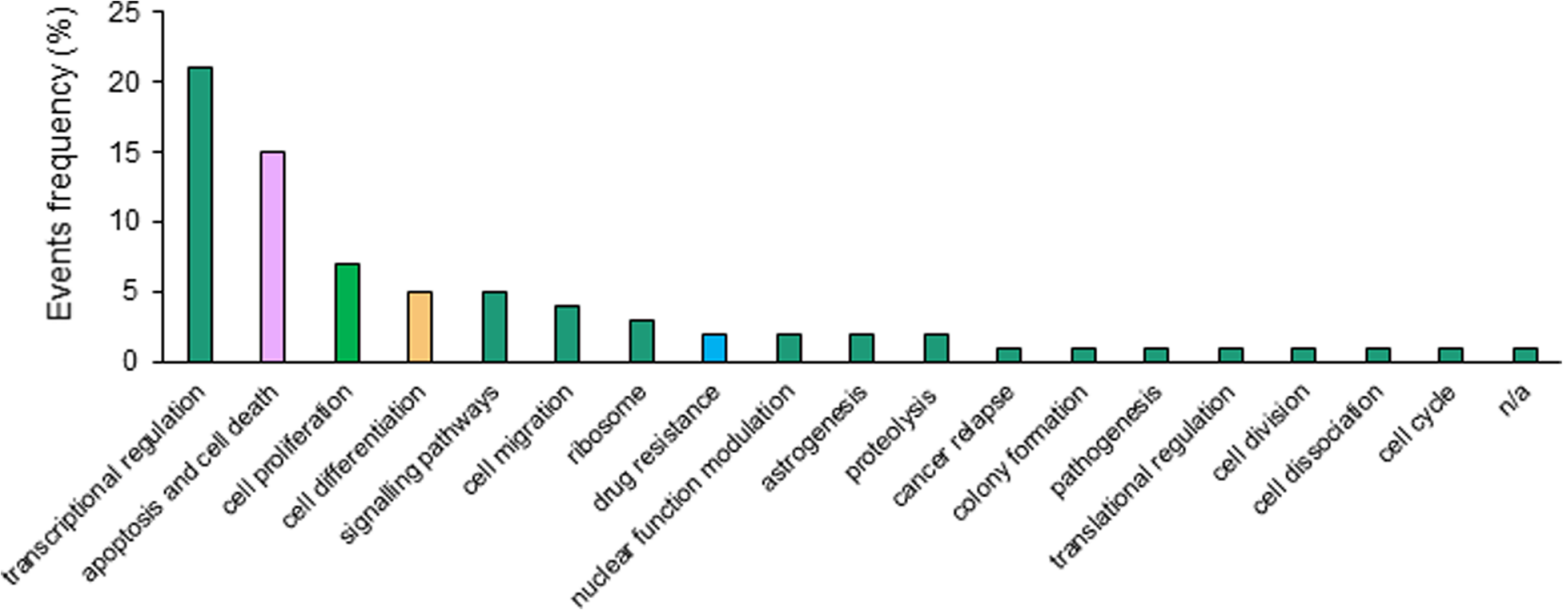
Functional analysis of nuclear localized membrane proteins in different biological activities using database. Functions of different nuclear localized membrane proteins were summarized using reported data. Nuclear localized membrane proteins were searched from the literatures of PubMed and google scholar and analyzed with biological functions

To gain insights into the main functions of nuclear localized membrane proteins (NLMPs) we searched database and found about 60 NLMPs, which have detailed references (Additional file 1: Table S1). We further summarized their functions through a database search. We found that dominant roles of these NLMPs are transcriptional regulation and cell death accounting for 21% and 15%, respectively, along with other highly ranked roles such as cell signaling pathways (5%), and drug resistance (2.5%) **(**Fig. 1 and Additional file 1: Table S1**)**. Thus, NLMPs possibly induce both cell death and drug resistance for survival. The paradoxical functions of NLMPs possibly fit Darwin’s theory of evolution and therapeutic survival induced by cancer evolution.

### Evolutionary origin of nuclear MET protein

**Fig. 2:**
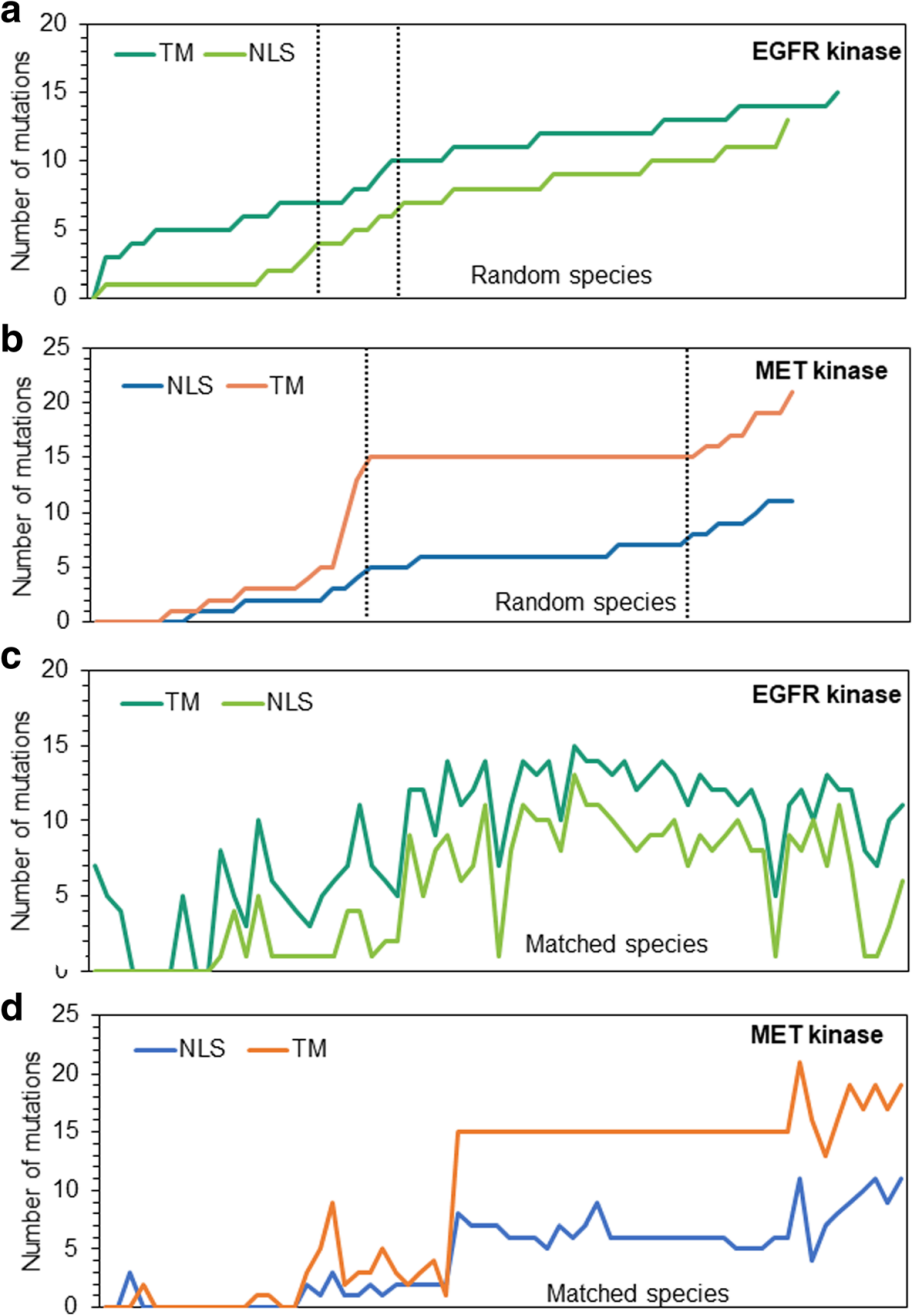
Phylogeneticly evolutionary analysis of nMET and nEGFR in different species. Alignment of sequences of nuclear localization signal (NLS) and transmembrane domain (TM) domain of EGFR (**a**, **c**) and MET (**b**, **d**) were analyzed and mutated sequences were counted and hit. The phylogenetic trees were constructed by methods described in main text using database [19–23]

Having elucidated nucleotide sequences of nMET and mMET proteins by using Uniprot database, Jalview and sequence alignment tools, we endeavored to uncover the evolutionary origin of nMET. For this purpose, we examined sequences that encode for nuclear localized signal (NLS) and transmembrane (TM) domain among 66 different species and tested the degree of point mutations in NLS and TM sequences from different animal species compared to human sequences. Using EGFR as control, we found that both NLS and TM of MET are conserved among species and showed parallel evolution based on overall mutation events in all species tested **(**Fig. 2a-d**)**. However, NLS undergoes more accelerated evolution than mMET **(**Fig. 2a-d**)**. Thus our data suggest that nMET may have been evolved from mMET, because of preserved stability of TM sequence in nearly 40% of all selected species. Moreover, nMET and mMET showed distinct interaction maps (data not shown), suggesting the different evolutionary paths of the two forms of MET.

### Nuclear MET induces both cell death and survival signaling

**Fig. 3:**
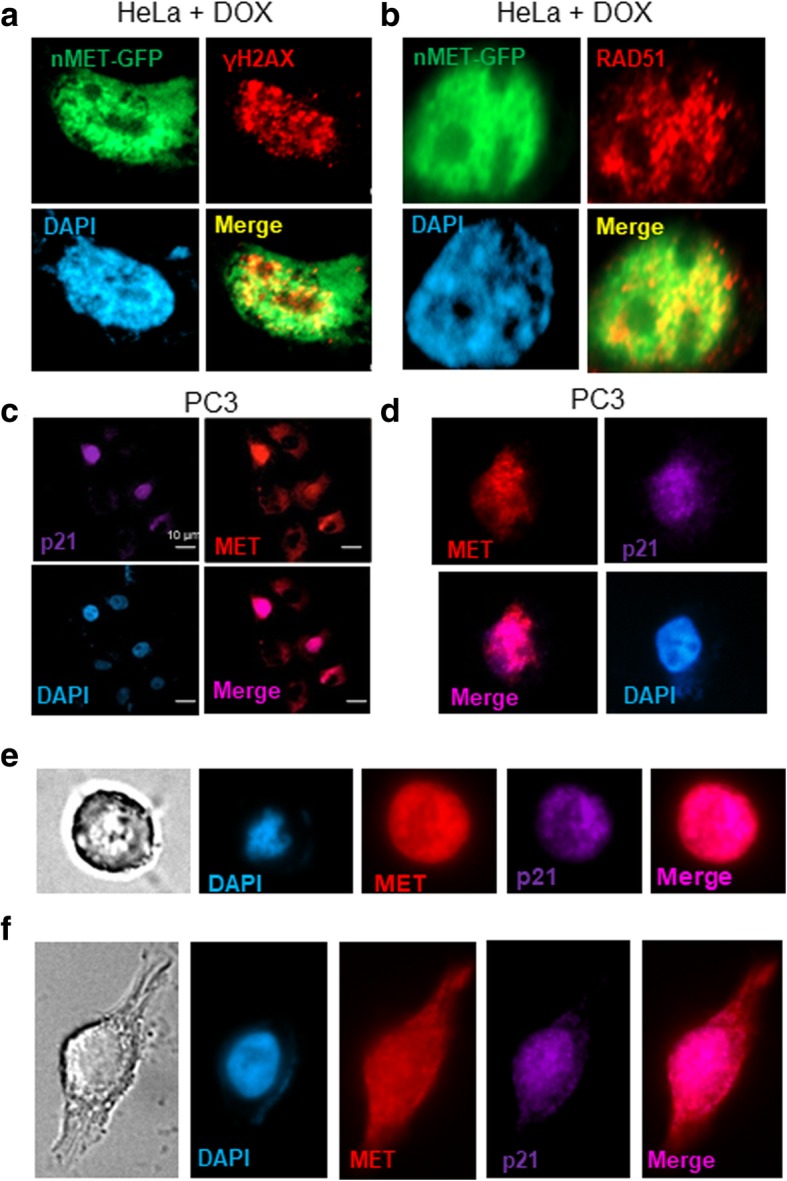
Nuclear MET associates with DNA damage and p21. **a**-**b** Nuclear MET of GFP fusion protein colocalizes with DNA damage and repair marker in HeLa cells upon drug treatment by doxorubicin (DOX). **c**-**d** Nuclear MET correlates and colocalizes with p21 in PC3 cells. **e**-**f** Nuclear MET associates with p21 in the dead cell or attached cell

**Fig. 4:**
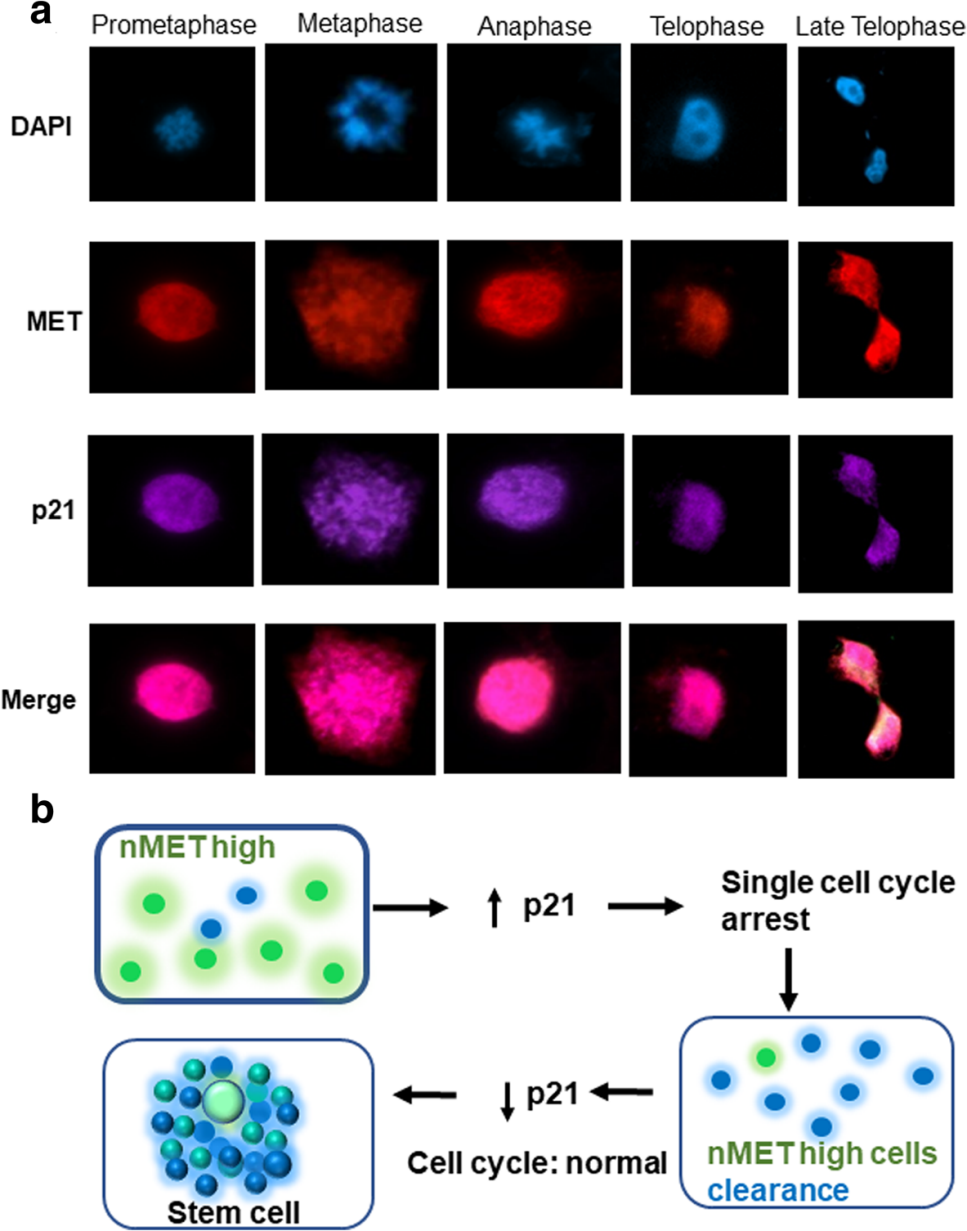
Nuclear MET associates with p21 in cell cycle of single cells. **a** PC3 cells were immunostained with anti-p21, anti-MET antibodies and DAPI. Cell cycle undergoing cells were listed in differential phases. **b** A proposed summary and model that nMET induced p21 and cell self-clearance may not affect whole cell cycle of population but single cells may evolve via reprograming or be selected as a cancer stem cell for survival

**Fig. 5:**
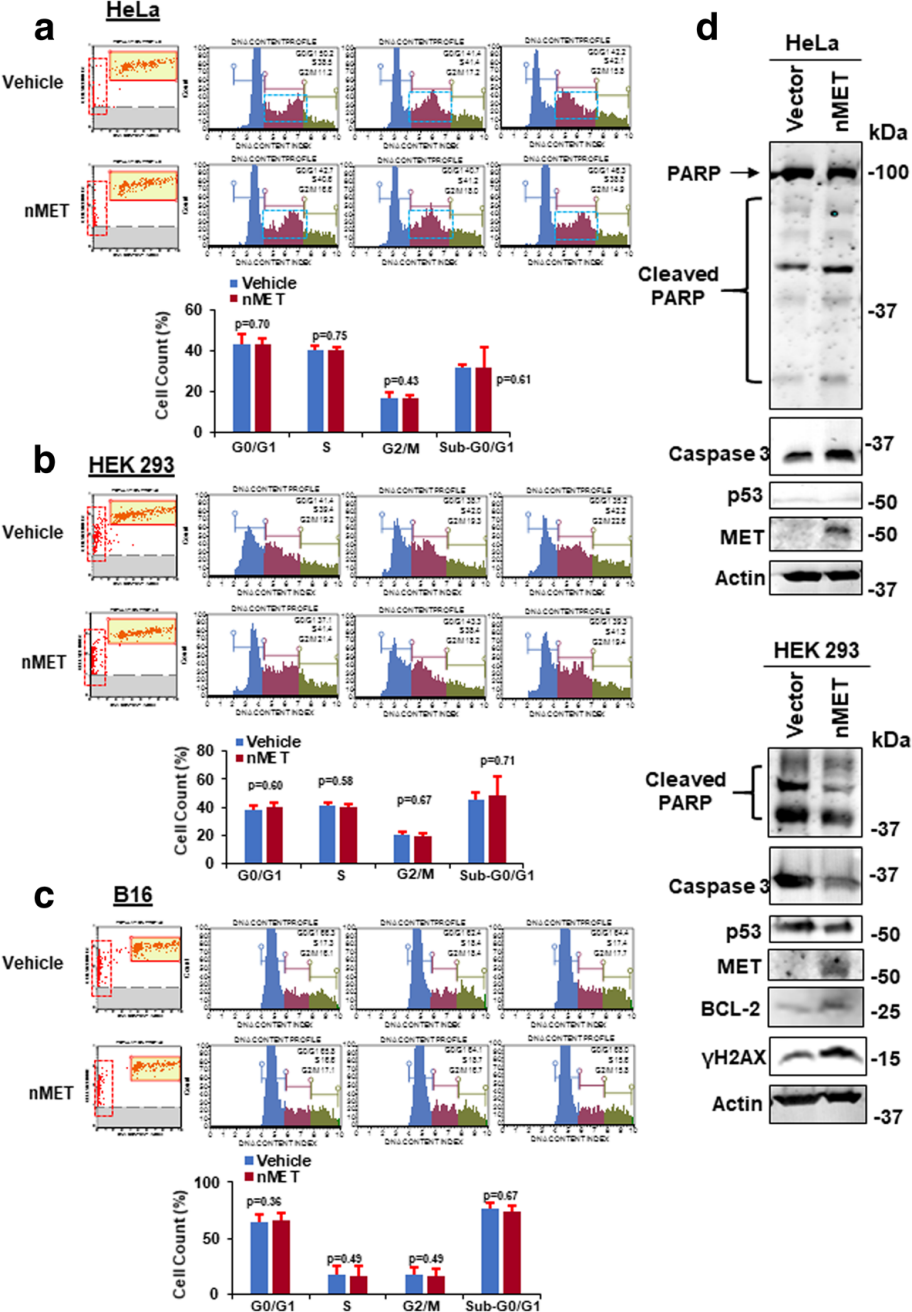
Effect of nuclear MET overexpression on cell cycle, cell death and survival signaling. **a**-**c** Effect of nuclear MET elevation on cell cycle by flow cytometry analysis. Cells indicated were transfected by plasmid containing CMV promoter-*nMET* gene and cell cycles were analyzed by DNA content. **d** Nuclear MET overexpression induces cell death and survival proteins in HeLa and HEK293 cells by western blot

To test whether nMET also paradoxically induces both cell death and survival, we examined the association between nMET and cell survival signaling pathways including apoptosis, anti-apoptosis, DNA damage and DNA repair in two cell lines, HeLa and HEK293. As shown in Fig. 3a-d, in HeLa cells treated with Doxorubicin (Dox) at 100 nM, nMET colocalizes with DNA damage marker γH2AX and DNA repair protein RAD51. In addition, endogenous nMET correlates with p21 expression (Fig. 3e, f). Importantly, nMET high cells showed round shape with high levels of p21 expression and detached trend which may undergo cell death (Fig. 3e, f). Furthermore, cell cycle analysis with high levels of endogenous nMET expressing individual cells showed that nMET may mediate cell cycle arrest in prostate cancer PC3 cells (Fig. 4a). While the potential cell death induced clearance of nMET highly expressed cells, may balance the overall cell population, to resistant the changes in the cell cycle of the whole population. Stemness may be the results of selection and clearance induced by nMET (Fig. 4b**)**. To test the hypothesis, we counted the total population of cell cycle by DNA content with flow cytometer. We found that overall nMET overexpression did not induce whole population changes in cell cycle but for single cells, the dynamics in DNA content distribution patterns were slightly different upon nMET overexpression (Fig. 5a-c). The dead cell populations also showed different patterns in scatter graphs of cell cycle analysis (Fig. 5a-c) in three cell lines we tested. Thus our data suggest that subsets of cells overexpressing nMET may undergo cell cycle arrest with quick clearance and the overall population of cells have not been undergone changes in cell cycle. Few number of nMET expressing survived cells may undergo evolution.

Next, to further test our hypothesis, we investigated levels of cell death and survival proteins in nMET overexpressed cells. As shown in Fig. 5d, nMET overexpressed cells showed higher or lower levels of cleaved Caspase 3, increased DNA damage marker γH2AX but also increased survival protein Bcl-2, dysregulated p53 and dysregulated cleavage of PARP. The paradoxical dysregulation of cell death and survival may suggest that nMET expressing cells may undergo clearance and survival for cell dynamic transformation. Thus our data suggest that nMET induces both cell death and cell survival signaling. Moreover, cell cycle arrest associated with nMET overexpression may be essential to the dysregulation of the cell death and survival for cells repopulation and evolution.

### Nuclear MET drives drug resistance and stemness for cell survival in subsets of cells

**Fig. 6:**
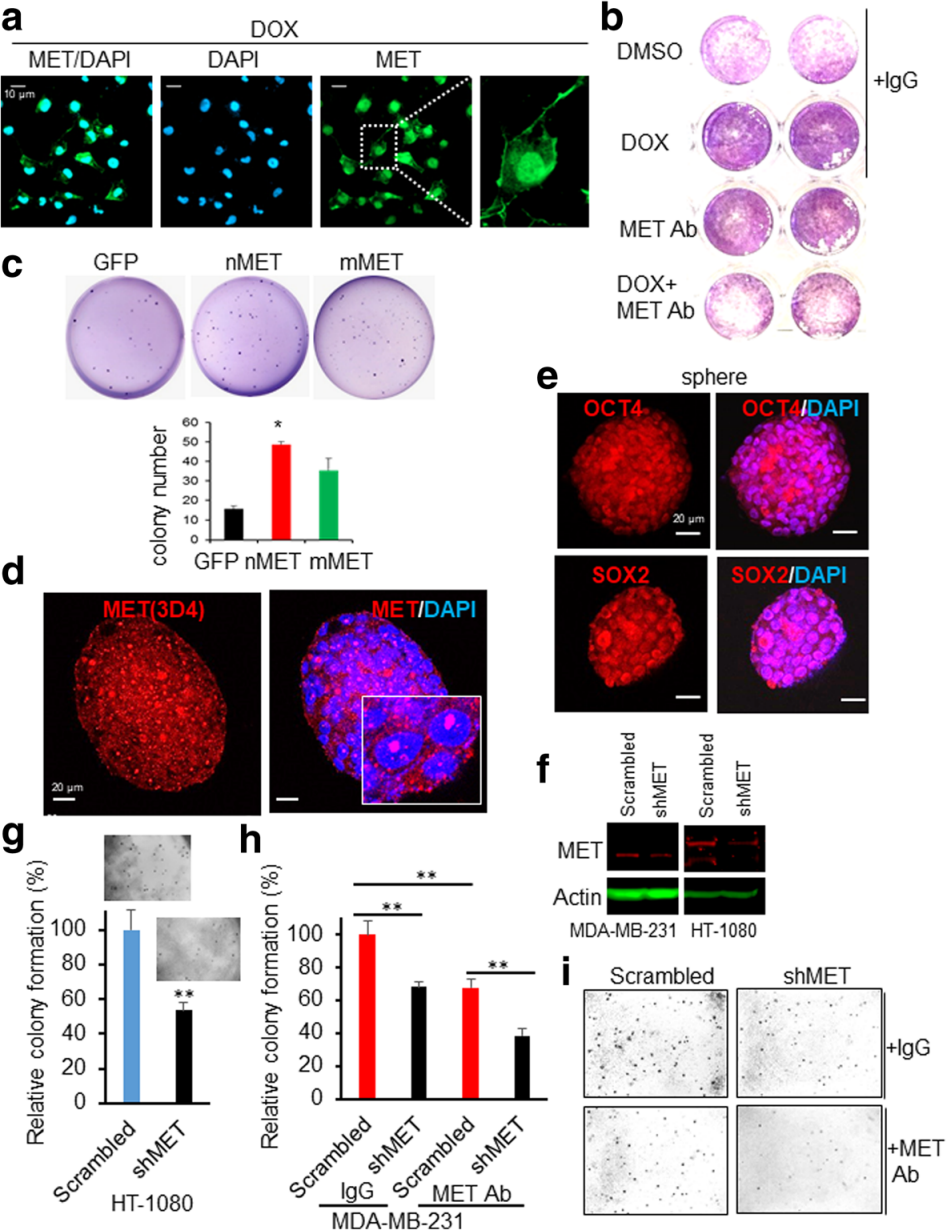
Nuclear MET mediates stemness and drug resistance. **a** Nuclear MET expression in PC3 cells upon drug response to doxorubicin (DOX). **b** Breast cancer MCF7 cells cytotoxicity assay upon treatment with DMSO (control), 60 nM doxorubicin (DOX) alone, antibody (Ab) against MET alone and combined treatment with Dox and antibody against MET. **c** Nuclear MET induces stem-like cell growth by colony formation assay. **d** Nuclear MET expression in stem-like cells of C4-2B formed sphere. **e** C4-2B formed spheres express stem cell markers of SOX2 and OCT4. **f**-**i** MET knockdown decreases cancer cell colony formation and membrane MET inhibition by MET antibody (MET Ab) further decreases colony formation

To understand how nMET might mediate drug resistance, we first tested the effect of Dox on cell survival (Fig. 6a-b). We first treated PC3 prostate cancer cells with the drug for 24 h. As shown in Fig. 6a, MET was localized in the nucleus upon drug treatment. Surprisingly, MCF7 breast cancer cells survived upon treatment with Dox, but Dox became effective when cells were treated with the antibody against MET (Fig. 6b). Thus our data suggest that drug resistance may allow clearance of nMET positive cells while survived cells might be nMET overexpressing cells which may have been undergone evolution.

To further test whether nMET is involved in stem cell-mediated evolution for drug resistance in survival, we first examined the potential of nMET and mMET in colony forming ability, a character of cancer-stem like cells. We found higher number of colonies in nMET overexpressed C4-2B cells compared to vector control and mMET transformed cells **(**Fig. 6c**)**. Next, we found moderate expression levels of endogenous nMET in prostate spheres formed by androgen receptor (AR)-insensitive cells of C4-2B cell line but not in 2-D cell culture condition (Fig. 6d and reference [14]). Spheres of C4-2B also exhibited stem cell-like properties expressing stem cell markers OCT4 and SOX2 **(**Fig. 6e**)**. Given that CSCs have characteristics of anti-cancer drug-mediated survival [18], high expression levels of nMET in spherical shape are in line with the potential role of nMET in drug resistance and survival through stemness. Finally, knockdown of MET decreased cancer cell colony formation, and upon additional inhibition of membrane MET by cell surface recognized MET antibody (Abcam), combined with knockdown of MET indicating predominantly nMET downregulation by knockdown, decreased colony formation efficiency significantly (Fig. 6f-i). Collectively, our data suggest that nMET might be essential in mediating drug resistance, and transformation which is in agreement with our previous finding that nMET mediates cancer stem-like cell self-renewal to promote cancer recurrence [14].

### Nuclear receptor tyrosine kinases correlate with poor prognosis based on database search and reanalysis

**Fig. 7:**
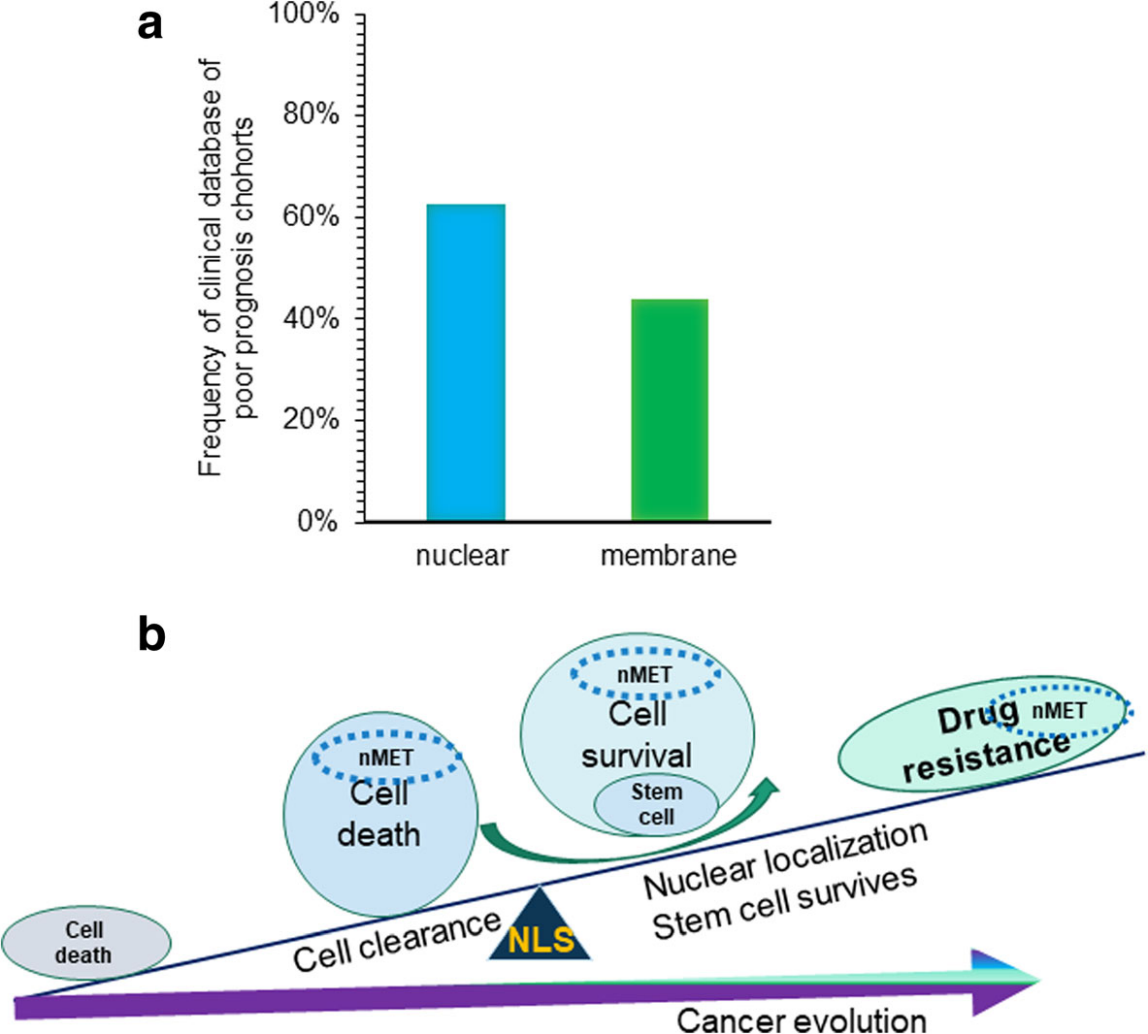
Nuclear receptor tyrosine kinases mediate poor prognosis based on database search and analysis. **a** The counted hits of reported clinical cohort studies were analyzed and most reported cases suggested the poor prognosis of nuclear localized RTKs compared to membrane RTKs. **b** A proposed model of nuclear RTK may through nuclear localization to clear unfitted dead cell to maintain membrane MET survival but may allow stem-like cells evolved to advanced recurrent cancer

To further investigate the correlation of nuclear RTK with the drug resistance, advanced cancer or prognosis, we searched database and summarized the results of published cohort studies. As shown in Fig. 7a and Additional file 1: Table S2, many studies have shown that RTK inhibitor or other types of drug resistance is mediated by crosstalk pathways even between RTK members. This finding may suggest more crosstalk in RTK evolution through TM and NLS. Most importantly, many studies suggest that nuclear RTK correlates with drug resistance, or decreased survival (Additional file 1: Table S2 and their references). Based on the counting and hits from individual studies, nuclear RTK may represent the poor prognosis in cancer patient survival. In summary, database analysis and our in vitro experimental data suggest that nuclear RTK may resemble the cancer evolution from cell death, clearance, and fitted survival through stemness (Fig. 7b).

## Discussion

Cumulative data from different sources demonstrate that a majority of membrane-bound NLMP proteins are involved in transcriptional regulation, apoptosis, cell migration, and drug resistance (Fig. 1**)**. In our study we focused on MET, a receptor tyrosine kinase family protein, which was reported to have two different forms in cells depending on cellular context – transmembrane and nuclear forms with a nuclear localization signal. In normal tissues following native ligand binding of hepatocyte growth factor, membranous MET regulates sensory neuron development, morphogenesis, embryogenesis, tissue regeneration and wound repair [48, 49]. Nuclear localization of MET is found in many cancer types, for instance, melanoma, breast, hepatocellular, and prostate carcinomas suggesting a more complex and multifunctional role of MET in oncogenesis [50–52]. In our study we proposed a model of the paradoxical functions of nMET in cancer cell death for clearance of mislocalized MET to sustain membrane MET function, and meanwhile, for survival, cancer stem cells may be the driver for aggressively evolved cancer through cancer stemness and differentiation.

Our experimental data demonstrated the association of the nMET with elevated expression of DNA damage and DNA repair-associated cellular biomarkers, γH2AX and RAD51 which are crucial molecular players in further induction of apoptosis. This finding suggests nMET is critical in regulation of the cell death. This is consistent with other reports that under certain stress stimuli C-terminal cleavage of cytoplasmic fragment of MET leads to apoptosis in epithelial cells [53, 54]. Our experimental results demonstrate that cells expressing high levels of nMET showed elevated expression of death signaling but also elevated Bcl-2 for survival. Moreover, endogenous nMET correlates with expression and colocalizes with an inhibitor of cell cycle, p21. Additionally, elevated expression levels of p21 and nMET were found to be linked to round morphology in cells which is typical of apoptotic cells. These results along with others further prove that nMET plays essential roles in cancer cell death and survival. To date, it is not known how nuclear RTK might function to regulate both cell survival and cell death in cancerous cells. There are many molecular switch mechanisms such as post-translational modifications including phosphorylation which may reverse the functions of signaling target. PTEN/AKT is one such switching pathway [55] which acts via phosphorylation and/or dephosphorylation of target molecules. In our previous report [56] we and our collaborators demonstrated that AKT is inhibited by MET inhibitor as a downstream target of MET in prostate cancer mouse model and cell lines. Thus AKT may be a switch to determine cell fate as death or survival. Further experiments are expected to explore the downstream effects of nMET and crosstalk with AKT pathways. Our previous reports suggest nMET is a phosphorylated form [14]. However, nMET is also reported as non-phosphorylated form [54]. One explanation might be the differential cleaved forms through different sites in different studies as truncated forms of nMET still contain kinase domain but kinase activity depends on the cleavage sites to maintain the intact of kinase domain.

Studies showed that MET, either full-length or cleaved MET may localize into cell nucleus by various mechanisms and under different cellular states and conditions [10, 51, 52]. In light of these observations we conducted a number of experiments to investigate the function of two different forms of MET. Our experimental data indicates that in PC3 cells MET is localized in the nucleus in response to treatment with doxorubicin. Our more data suggest that extracellular stress may promote MET nuclear translocation to regulate DNA damage, enhance DNA repair to prevent cell death. A number of mechanisms proposed by other groups show drug resistance acquirement by cancer cells [5, 57, 58]. However, of particular importance are tumor-initiating/ stem-like cells which are essential for the castration-resistant prostate cancer and other cancer types of recurrence [59, 60]. Nowadays, androgen deprivation therapy is routinely used to treat prostate cancer. However, there has been a rise in castration-resistant cancer in patients treated with this type of therapy. This phenomenon might be responsible for development of the cancer stem-like cells under selective pressure of androgen deprivation. Our experimental results revealed that C4-2B cells form sphere-like structures which are characteristics of stem cells and are resistant to doxorubicin treatment. Further analysis showed that these cells express OCT4 and SOX2 transcription factors which are typically associated with embryonic stem cells pluripotent potential and self-renewal [61]. Using immunofluorescent staining we found localization of MET in the nucleus of stem-like prostate spheres. Our data suggest a function of nMET in cancer stem-like cell induced drug resistance. Thus MET is most likely involved in mediating therapy resistance in cancer cells through promoting survival of cells exhibiting stem cell-like properties. Such mechanism further suggests that nMET might also promote cancer cell evolution when cells are subjected to selective pressures such as anticancer drugs. More research is needed to elucidate exact mechanisms of regulation of these dynamic processes. It is still not clear how nMET induces cancer stem cells. Based on our recent findings on MMP family protein evolution and translocation, more studies are needed to elucidate both NLMP and disease evolutions [62]. Recently we found YAP is also a shuttling protein localized in cytosol, nucleus and membrane and is regulated by nuclear protein ARF [63]. In addition, MMP nuclear localization correlates to ARF elevation in prostate cancer cells [64]. However, whether oncogenic-like ARF regulates NLMP during cancer progression as a general mechanism remains elusive [65]. Thus, more research is required to fully comprehend complex regulations and interactions that may occur between NLMP/nMET and other molecules in the nucleus. This would be the direction of our future research along with further investigations of dynamics of evolved NLMP/nRTK in drug resistance and stemness during cell clearance.

## Conclusions

This is the first conceptual link between nuclear RTK/MET kinases to cancer evolution and clinical investigation including cancer stem-like cells in drug adaption and resistant survival which fits the Darwin theory. This link resolved the paradox on both cell death and survival in heterogenesis of cancer cell evolution and recurrence.

## Additional file

Additional file 1: **Table S1.** Nuclear localized membrane proteins were searched from the literatures of PubMed and google scholar and analyzed with biological functions. **Table S2.** Drug resistance pathways and survival rate dependence on nuclear localization in RTKs. Thistable reveals different receptor tyrosine kinase (RTK) families and pathways by which they induce drug resistance. The RTKs with localization to nucleus are indicated with “+” sign. The survival rate of patients was assessed by using PubMed database, where we entered the following keywords as “nuclear translocation/localisationof in patients, clinical data”. This allowed us to observe the relation between the translocation of proteins and survival rate. NA states for the absence of evidence regarding particular criteria. (PDF 338 kb)

## Abbreviations

ATCC: The American Type Culture Collection
mMET: Membrane MET
NLMP: Nuclear localized membrane protein
NLS: Nuclear localization signal
nMET: Nuclear MET
RTK: Receptor tyrosine kinase
