# Supplementary material for: Systematic analysis of NLMP suggests nuclear localization of RTK/MET kinases resemble cancer cell clearance
Source: J Exp Clin Cancer Res. 2019 Jan 30;38:43. doi: 10.1186/s13046-018-1004-z (PMC6354337; doi:10.1186/s13046-018-1004-z)
Supplement: Supplementary file 1 — Table S1. Nuclear localized membrane proteins were searched from the literatures of PubMed and google scholar and analyzed with biological functions. Table S2. Drug resistance pathways and survival rate dependence on nuclear localization in RTKs. Thistable reveals different receptor tyrosine kinase (RTK) families and pathways by which they induce drug resistance. The RTKs with localization to nucleus are indicated with “+” sign. The survival rate of patients was assessed by using PubMed database, where we entered the following keywords as “nuclear translocation/localisationof in patients, clinical data”. This allowed us to observe the relation between the translocation of proteins and survival rate. NA states for the absence of evidence regarding particular criteria. (PDF 338 kb) [file 13046_2018_1004_MOESM1_ESM.pdf]

Supplementary data for

Systematic analysis of NLMP suggests nuclear localization of RTK/MET kinases resemble cancer cell clearance

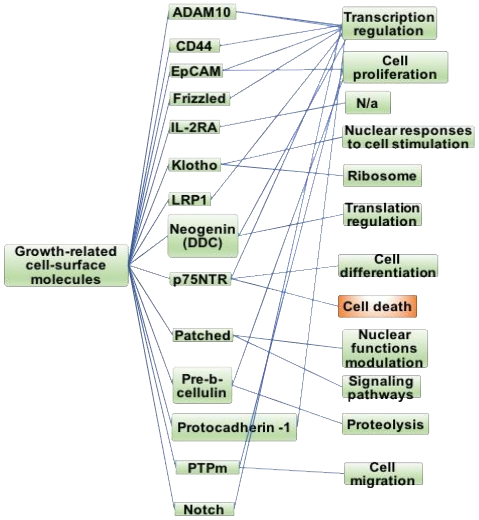

Supplementary table 1 Nuclear localized membrane proteins were searched from the literatures of PubMed and google scholar and analyzed with biological functions.

RTK family Name	Drug resistance tested pathway	Nuclear localization	Reference	Poorer Patients' Survival (nuclear vs membrane)	Reference
**ErbB (spec. EGFR)**	Akt, MET	+	(Jacobsen et al., 2017)	Nuclear	(Lo et al., 2005)
	Ras, c-Met, HER2, IGF1R, FGFR1		(Xu et al., 2017)		
**Insulin**	Akt	+	(Qi et al., 2018)	Nuclear	(Aleksic et al., 2010)
	P38MAPK/PGC-1α/GLUT4		(Penghua et al., 2018)		
**PDGF**	PI3K/Akt	+	(Ding et al., 2010)	NA	-
**VEGF**	PI3K/Akt/mTOR/HIF1alpha	+	(Jui-Ling, et al.. 2016)	Nuclear	(El-Chemaly et al., 2014)
**FGF**	TYK2, Erk1/2, Bcl-2	+	(Carmo et al., 2011)	Nuclear	(Zhou et al., 2015)
**PTK7**	Wnt, Akt	+	(Berger et al., 2017)	Nuclear	(Ataseven et al., 2013)
**Trk**	PKC, Ras/MAPK/PI3	+	(Bonacchi et al., 2008 Jang et al., 2017)	Membrane	(Ray et al., 2004)
**Ror**	NF-kB, TGF-B,LXR	+	(Ror et al., 2009)	Nuclear	(Rebagay et al., 2012)
**MuSK**	Dok-7, Rac, Rho	-	(Burden et al., 2013)	Membrane	(Chen et al., 2017)
**Met**	GRB2, HGF, TGF-b	+	(Li et al., 2017 Sun et al., 2017)	Nuclear	(Pectasides et. al., 2014)
**Axl**	PI3K/Akt, Src/MAPK/ERK pathways	+	(Lu et al., 2016)	Membrane	(Gay,Balaji and Byers, 2017)
**Tie**	Ang-1/Tie-2 signaling pathway	+	(Carpenter & Liao,2013 Tang et al.,2016)	Membrane	(Tang et al.,2016)
**Eph**	HRAS-Erk, PI3 kinase-Akt and Abl-Crk pathways	+	(Pasquale, 2010)	Nuclear	(Husa et al.,2016)
**Ret**	ERK and AKT	+	(Bagheri-Yarmand et al.,2015)	Membrane	(Chang et al., 2017)
**Ryk**	Wnt	+	(Carpenter & Liao, 2013)	Nuclear	(Green et al., 2014)
**DDR Discoidin Domain Receptors (DDRs 1 and 2)**	DDR1: Ras/Raf/ERK and protein kinase C dependent pathways; DDR2: phospho-JAK2 recruitment to DDR2 and ERK1/2 activation,Wnt5a/Frizzled, Notch1	+	(Valiathan et al., 2012)	NA	(Ford et al., 2007) (Toy et al., 2015)
**Ros**	STAT3 PI3K/AKT/m-TOR	+	(Cheng et al., 2015)	Equal	(Cheng et al., 2015)
**ALK anaplastic lymphoma kinase**	phospholipase Cγ, JAK/STAT, PI3K-AKT, mTOR, sonic hedgehog, JUNB, CRKL-C3G,RAP1 GTPase and MAPK	NA	(Reshetnyak et al. 2015) (Zhao Z et al., 2015)	NA	(Liu et al. 2016)
**STYK1**	MEK/ERK and PI3K/AKT pathway	NA	(Zhao, 2017)	Membrane	(Wang et al., 2016)

Supplementary table 2: Drug resistance pathways and survival rate dependence on nuclear localization in RTKs. This table reveals different receptor tyrosine kinase (RTK) families and pathways by which they induce drug resistance. The RTKs with localization to nucleus are indicated with "+" sign. The survival rate of patients was assessed by using PubMed database, where we entered the following keywords as "nuclear translocation/localisation of … in patients, clinical data". This allowed us to observe the relation between the translocation of proteins and survival rate. NA states for the absence of evidence regarding particular criteria.

References (Relates to Fig 1, tableS1, Fig 7, and Table S2):

1.Carpenter G, Liao H-J. Trafficking of receptor tyrosine kinases to the nucleus. *Experimental Cell Research*2009; **315**: 1556–1566.
2.Lin K-T, Sloniowski S, Ethell DW, Ethell IM. Ephrin-B2-induced Cleavage of EphB2 Receptor Is Mediated by Matrix Metalloproteinases to Trigger Cell Repulsion. *Journal of Biological Chemistry*2008; **283**: 28969–28979.
3.Song S, Rosen KM, Corfas G. Biological function of nuclear receptor tyrosine kinase action. Cold Spring Harb Perspect Biol. 2013;5(7)
4.Bavelloni A, Piazzi M, Raffini M, Faenza I, Blalock WL. Prohibitin 2: At a communications crossroads. IUBMB Life. 2015;67(4):239-54.
5.Carpenter G, Liao HJ. Receptor tyrosine kinases in the nucleus. Cold Spring Harb Perspect Biol. 2013;5(10):a008979.
6.Reif R, Adawy A, Vartak N, et al. Activated ErbB3 Translocates to the Nucleus via Clathrin-independent Endocytosis, Which Is Associated with Proliferating Cells. J Biol Chem. 2016;291(8):3837-47.
7.Song S, Rosen KM, Corfas G. Biological function of nuclear receptor tyrosine kinase action. Cold Spring Harb Perspect Biol. 2013;5(7)
8.Cai H, Orwenyo J, Guenaga J, et al. Synthetic multivalent V3 glycopeptides display enhanced recognition by glycan-dependent HIV-1 broadly neutralizing antibodies. Chem Commun (Camb). 2017;53(39):5453-5456.
9.Zammit C, Barnard R, Gomm J, et al. Altered intracellular localization of fibroblast growth factor receptor 3 in human breast cancer. J Pathol. 2001;194(1):27-34.
10.Kasuga K, Kaneko H, Nishizawa M, Onodera O, Ikeuchi T. Generation of intracellular domain of insulin receptor tyrosine kinase by gamma-secretase. Biochem Biophys Res Commun. 2007;360(1):90-6.
11.Foveau B, Ancot F, Leroy C, et al. Down-regulation of the met receptor tyrosine kinase by presenilin-dependent regulated intramembrane proteolysis. Mol Biol Cell. 2009;20(9):2495-507.
12.Pozner-moulis S, Pappas DJ, Rimm DL. Met, the hepatocyte growth factor receptor, localizes to the nucleus in cells at low density. Cancer Res. 2006;66(16):7976-82.
13.Lyu J, Yamamoto V, Lu W. Cleavage of the Wnt receptor Ryk regulates neuronal differentiation during cortical neurogenesis. Dev Cell. 2008;15(5):773-80.
14.Kristeleit R, Shapiro GI, Burris HA, et al. A Phase I-II Study of the Oral Poly(ADP-ribose) Polymerase Inhibitor Rucaparib in Patients with Germline BRCA1/2-mutated Ovarian Carcinoma or Other Solid Tumors. Clin Cancer Res. 2017;
15.Tousseyn T, Thathiah A, Jorissen E, et al. ADAM10, the rate-limiting protease of regulated intramembrane proteolysis of Notch and other proteins, is processed by ADAMS-9, ADAMS-15, and the gamma-secretase. J Biol Chem. 2009;284(17):11738-47.
16.Arima T, Enokida H, Kubo H, et al. Nuclear translocation of ADAM-10 contributes to the pathogenesis and progression of human prostate cancer. Cancer Sci. 2007;98(11):1720-6.
17.Devisser A, Yang C, Herring A, Martinez JA, Rosales-Hernandez A, Poliakov I, Ayer A, Garven A, Zaver S, Rincon N, Xu K, Tuor UI, Schmidt AM, Toth C. Differential impact of diabetes and hypertension in the brain: adverse effects in grey matter. Neurobiol Dis. 2011 Nov;44(2):161-73. doi:10.1016/j.nbd.2011.06.005.
18.Janiszewska M, De vito C, Le bitoux MA, Fusco C, Stamenkovic I. Transportin regulates nuclear import of CD44. J Biol Chem. 2010;285(40):30548-57.
19.Denzel S, Maetzel D, Mack B, Eggert C, Bärr G, Gires O. Initial activation of EpCAM cleavage via cell-to-cell contact. BMC Cancer. 2009;9:402.
20.Mathew D, Ataman B, Chen J, Zhang Y, Cumberledge S, Budnik V. 2005. Wingless signaling at synapses is through cleavage and nuclear import of receptor DFrizzled2. Science 310: 1344–1347.
21.Montes de oca P, Malardé V, Proust R, Dautry-varsat A, Gesbert F. Ectodomain shedding of interleukin-2 receptor beta and generation of an intracellular functional fragment. J Biol Chem. 2010;285(29):22050-8.
22.German DC, Khobahy I, Pastor J, Kuro-o M, Liu X. Nuclear localization of Klotho in brain: an anti-aging protein. Neurobiol Aging. 2012;33(7):1483.e25-30.
23.Zurhove K, Nakajima C, Herz J, Bock HH, May P. Gamma-secretase limits the inflammatory response through the processing of LRP1. Sci Signal. 2008;1(47):ra15.
24.Goldschneider D, Rama N, Guix C, Mehlen P. The neogenin intracellular domain regulates gene transcription via nuclear translocation. Mol Cell Biol. 2008;28(12):4068-79.
25.Ceni C, Kommaddi RP, Thomas R, et al. The p75NTR intracellular domain generated by neurotrophin-induced receptor cleavage potentiates Trk signaling. J Cell Sci. 2010;123(Pt 13):2299-307.
26.Parkhurst CN, Zampieri N, Chao MV. Nuclear localization of the p75 neurotrophin receptor intracellular domain. J Biol Chem. 2010;285(8):5361-8.
27.Le moan N, Houslay DM, Christian F, Houslay MD, Akassoglou K. Oxygen-dependent cleavage of the p75 neurotrophin receptor triggers stabilization of HIF-1α. Mol Cell. 2011;44(3):476-90.
28.Kagawa H, Shino Y, Kobayashi D, et al. A novel signaling pathway mediated by the nuclear targeting of C-terminal fragments of mammalian Patched 1. PLoS ONE. 2011;6(4):e18638.
29.Stoeck A, Shang L, Dempsey PJ. Sequential and gamma-secretase-dependent processing of the betacellulin precursor generates a palmitoylated intracellular-domain fragment that inhibits cell growth. J Cell Sci. 2010;123(Pt 13):2319-31.
30.Buchanan SM, Schalm SS, Maniatis T. Proteolytic processing of protocadherin proteins requires endocytosis. Proc Natl Acad Sci USA. 2010;107(41):17774-9.
31.Burgoyne AM, Phillips-mason PJ, Burden-gulley SM, et al. Proteolytic cleavage of protein tyrosine phosphatase mu regulates glioblastoma cell migration. Cancer Res. 2009;69(17):6960-8.
32.Chandra M, Zang S, Li H, et al. Nuclear translocation of type I transforming growth factor β receptor confers a novel function in RNA processing. Mol Cell Biol. 2012;32(12):2183-95.
33.Bao J, Lin H, Ouyang Y, et al. Activity-dependent transcription regulation of PSD-95 by neuregulin-1 and Eos. Nat Neurosci. 2004;7(11):1250-8.
34.Anders L, Mertins P, Lammich S, et al. Furin-, ADAM 10-, and gamma-secretase-mediated cleavage of a receptor tyrosine phosphatase and regulation of beta-catenin's transcriptional activity. Mol Cell Biol. 2006;26(10):3917-34.
35.Haapasalo A, Kim DY, Carey BW, Turunen MK, Pettingell WH, Kovacs DM. Presenilin/gamma-secretase-mediated cleavage regulates association of leukocyte-common antigen-related (LAR) receptor tyrosine phosphatase with beta-catenin. J Biol Chem. 2007;282(12):9063-72.
36.May P, Reddy YK, Herz J. Proteolytic processing of low density lipoprotein receptor-related protein mediates regulated release of its intracellular domain. J Biol Chem. 2002;277(21):18736-43.
37.El fiky A, Arch AE, Krolewski JJ. Intracellular domain of the IFNaR2 interferon receptor subunit mediates transcription via Stat2. J Cell Physiol. 2005;204(2):567-73.
38.El fiky A, Pioli P, Azam A, Yoo K, Nastiuk KL, Krolewski JJ. Nuclear transit of the intracellular domain of the interferon receptor subunit IFNaR2 requires Stat2 and Irf9. Cell Signal. 2008;20(7):1400-8.
39.Gao Y, Pimplikar SW. The gamma -secretase-cleaved C-terminal fragment of amyloid precursor protein mediates signaling to the nucleus. Proc Natl Acad Sci USA. 2001;98(26):14979-84.
40.Becker-herman S, Arie G, Medvedovsky H, Kerem A, Shachar I. CD74 is a member of the regulated intramembrane proteolysis-processed protein family. Mol Biol Cell. 2005;16(11):5061-9.
41.Böhm C, Seibel NM, Henkel B, Steiner H, Haass C, Hampe W. SorLA signaling by regulated intramembrane proteolysis. J Biol Chem. 2006;281(21):14547-53.
42.Hiesberger T, Gourley E, Erickson A, et al. Proteolytic cleavage and nuclear translocation of fibrocystin is regulated by intracellular Ca2+ and activation of protein kinase C. J Biol Chem. 2006;281(45):34357-64.
43.Galichet A, Weibel M, Heizmann CW. Calcium-regulated intramembrane proteolysis of the RAGE receptor. Biochem Biophys Res Commun. 2008;370(1):1-5.
44.Goldschneider D, Rama N, Guix C, Mehlen P. The neogenin intracellular domain regulates gene transcription via nuclear translocation. Mol Cell Biol. 2008;28(12):4068-79.
45.Bonacchi A, Taddei ML, Petrai I, et al. Nuclear localization of TRK-A in liver cells. Histol Histopathol. 2008;23(3):327-40.
46.Zwaagstra JC, Guimond A, O'connor-mccourt MD. Predominant intracellular localization of the type I transforming growth factor-beta receptor and increased nuclear accumulation after growth arrest. Exp Cell Res. 2000;258(1):121-34.
47.Andersson MK, Göransson M, Olofsson A, Andersson C, Aman P. Nuclear expression of FLT1 and its ligand PGF in FUS-DDIT3 carrying myxoid liposarcomas suggests the existence of an intracrine signaling loop. BMC Cancer. 2010;10:249.

48. Lemmon MA, Schlessinger J. Cell Signaling by Receptor Tyrosine Kinases. *Cell*2010; 141: 1117–1134.
49.Jacobsen K, Bertran-Alamillo J, Molina MA, Teixidó C, Karachaliou N, Pedersen MH *et al.*Convergent Akt activation drives acquired EGFR inhibitor resistance in lung cancer. *Nature Communications* 2017; 8. doi:10.1038/s41467-017-00450-6.
50.Xu J, Wang J, Zhang S. Mechanisms of resistance to irreversible epidermal growth factor receptor tyrosine kinase inhibitors and therapeutic strategies in non-small cell lung cancer. *Oncotarget*2017. doi:10.18632/oncotarget.21164.
51.Xu Q, Luo J, Wu N, Zhang R, Shi D. BPN, a marine-derived PTP1B inhibitor, activates insulin signaling and improves insulin resistance in C2C12 myotubes. *International Journal of Biological Macromolecules*2018; 106: 379–386.
52.Fang P, Zhang L, Yu M, Sheng Z, Shi M, Zhu Y *et al.* Activiated galanin receptor attenuates insulin resistance in skeletal muscle of obese mice. *Peptides* 2018; 99: 92–98.
53.Lo HW, Xia W, Wei Y, Ali-Seyed M, Huang SF, Hung MC. Novel prognostic value of nuclear epidermal growth factor receptor in breast cancer. Cancer Res. 2005b;65:338–348.
54.Aleksic T, Chitnis MM, Perestenko OV, Gao S, Thomas PH, Turner GD *et al.* Type 1 Insulin-like Growth Factor Receptor Translocates to the Nucleus of Human Tumor Cells. *Cancer Research* 2010; 70: 6412–6419.
55.Ding W, Knox TR, Tschumper RC, Wu W, Schwager SM, Boysen JC *et al.* Platelet-derived growth factor (PDGF)-PDGF receptor interaction activates bone marrow-derived mesenchymal stromal cells derived from chronic lymphocytic leukemia: implications for an angiogenic switch. *Blood* 2010; 116: 2984–2993.
56.Tsai J-L, Lee Y-M, Pan C-Y, Lee AY-L. The Novel VEGF121-VEGF165 Fusion Attenuates Angiogenesis and Drug Resistance via Targeting VEGFR2-HIF-1α-VEGF165/Lon Signaling Through PI3K-AKT-mTOR Pathway. *Current Cancer Drug Targets* 2016; 16: 275–286.
57.El-Chemaly S, Pacheco-Rodriguez G, Malide D, Meza-Carmen V, Kato J, Cui Y *et al.* Nuclear Localization of Vascular Endothelial Growth Factor-D and Regulation of c-Myc–Dependent Transcripts in Human Lung Fibroblasts. *American Journal of Respiratory Cell and Molecular Biology* 2014; 51: 34–42.
58.Carmo CR, Lyons-Lewis J, Seckl MJ, Costa-Pereira AP. A Novel Requirement for Janus Kinases as Mediators of Drug Resistance Induced by Fibroblast Growth Factor-2 in Human Cancer Cells. *PLoS ONE*2011; 6. doi:10.1371/journal.pone.0019861.
59.Zhou L, Yao L-T, Liang Z-Y, et al. Nuclear translocation of fibroblast growth factor receptor 3 and its significance in pancreatic cancer. *International Journal of Clinical and Experimental Pathology*. 2015;8(11):14640-14648.
60.Pectasides E, Rampias T, Sasaki C, Perisanidis C, Kouloulias V, Burtness B *et al.*Markers of Epithelial to Mesenchymal Transition in Association with Survival in Head and Neck Squamous Cell Carcinoma (HNSCC). *PLoS ONE*2014; 9. doi:10.1371/journal.pone.0094273.
61.Li W, Zhang X, Wang J, Li M, Cao C, Tan J *et al.*TGF betta in fibroblasts-derived exosomes promotes epithelial-mesenchymal transition of ovarian cancer cells. *Oncotarget*2017. doi:10.18632/oncotarget.21635.
62.Sun Z, Liu Q, Ye D, Ye K, Yang Z, Li D. Role of c-Met in the progression of human oral squamous cell carcinoma and its potential as a therapeutic target. *Oncology Reports*2017. doi:10.3892/or.2017.6073.
63.Ray A. A Clinicobiological Model Predicting Survival in Medulloblastoma. *Clinical Cancer Research*2004; 10: 7613–7620.
64.Bonacchi A, Taddei L, Efsen E, Defranco R, Nosi D, Petrai I *et al.*Nuclear localization of the nerve growth factor (NGF) receptor, TRK-A in liver cells. *Journal of Hepatology*2003; 38: 75.
65.Jang J-Y, Hong YJ, Lim J, Choi JS, Choi EH, Kang S *et al.*Cold atmospheric plasma (CAP), a novel physicochemical source, induces neural differentiation through cross-talk between the specific RONS cascade and Trk/Ras/ERK signaling pathway. *Biomaterials*2017. doi:10.1016/j.biomaterials.2017.11.045.
66.André MDR, Amaral S, Mayer A, Miranda A, Sul WGR. Breast Cancer Patients Survival and Associated Factors: Reported Outcomes from the Southern Cancer Registry in Portugal. *Acta Médica Portuguesa*2014; 27: 325.
67.Jetten. Retinoid-related orphan receptors (RORs): critical roles in development, immunity, circadian rhythm, and cellular metabolism. *Nuclear Receptor Signaling*2007; 4. doi:10.1621/nrs.07003.
68.Ataseven B, Angerer R, Kates R, Gunesch A, Knyazev P, Högel B, Becker C, Eiermann W, Harbeck N. PTK7 expression in triple-negative breast cancer. *Anticancer Res.* 2013 Sep;33(9):3759-63
69.Berger H, Breuer M, Peradziryi H, Podleschny M, Jacob R, Borchers A. PTK7 localization and protein stability is affected by canonical Wnt ligands. *Journal of Cell Science*2017; 130: 1890–1903.
70.Chen G, Yu H, Satherley L, Zabkiewicz C, Resaul J, Zhao H *et al.*The downstream of tyrosine kinase 7 is reduced in lung cancer and is associated with poor survival of patients with lung cancer. *Oncology Reports*2017; 37: 2695–2701.
71.Burden SJ, Yumoto N, Zhang W. The Role of MuSK in Synapse Formation and Neuromuscular Disease. *Cold Spring Harbor Perspectives in Biology*2013; 5. doi:10.1101/cshperspect.a009167.
72.Valiathan RR, Marco M, Leitinger B, Kleer CG, Fridman R. Discoidin domain receptor tyrosine kinases: new players in cancer progression. *Cancer and Metastasis Reviews*2012; 31: 295–321.
73.Ford CE, Lau SK, Zhu CQ, Andersson T, Tsao MS, Vogel WF. Expression and mutation analysis of the discoidin domain receptors 1 and 2 in non-small cell lung carcinoma. *British Journal of Cancer*2007; 96: 808–814.
74.Toy KA, Valiathan RR, Núñez F, Kidwell KM, Gonzalez ME, Fridman R *et al.*Tyrosine kinase discoidin domain receptors DDR1 and DDR2 are coordinately deregulated in triple-negative breast cancer. *Breast Cancer Research and Treatment*2015; 150: 9–18.
75.El-Deeb IM, Yoo KH, Lee SH. ROS receptor tyrosine kinase: a new potential target for anticancer drugs. *Medicinal Research Reviews*2010. doi:10.1002/med.20206.
76.Cheng Y, Sun Y, Wang LZ, Yu YC, Ding X. Cytoplasmic c-ros oncogene 1 receptor tyrosine kinase expression may be associated with the development of human oral squamous cell carcinoma. *Oncology Letters*2015. doi:10.3892/ol.2015.3340.
77.Reshetnyak AV, Murray PB, Shi X, Mo ES, Mohanty J, Tome F *et al.*Augmentor α and β (FAM150) are ligands of the receptor tyrosine kinases ALK and LTK: Hierarchy and specificity of ligand–receptor interactions. *Proceedings of the National Academy of Sciences*2015; 112: 15862–15867.
78.Zhao Z, Verma V, Zhang M. Anaplastic lymphoma kinase: Role in cancer and therapy perspective. *Cancer Biology & Therapy*2015; 16: 1691–1701.
79.Liu J, Jin H, Tian H, Lian G, Chen S, Li J *et al.*Anaplastic lymphoma kinase protein expression predicts micrometastases and prognosis for patients with hepatocellular carcinoma. *Oncology Letters*2015. doi:10.3892/ol.2015.3859.
80.Zhao Y, Yang L, He J, Yang H. STYK1 promotes Warburg effect through PI3K/AKT signaling and predicts a poor prognosis in nasopharyngeal carcinoma. *Tumor Biology*2017; 39: 101042831771164.
81.Wang Z, Qu L, Deng B, Sun X, Wu S, Liao J *et al.*STYK1 promotes epithelial-mesenchymal transition and tumor metastasis in human hepatocellular carcinoma through MEK/ERK and PI3K/AKT signaling. *Scientific Reports*2016; 6. doi:10.1038/srep33205.
82.Gay CM, Balaji K, Byers LA. Giving AXL the axe: targeting AXL in human malignancy. *British Journal of Cancer*2017; 116: 415–423.
83.Lu Y, Wan J, Yang Z, Lei X, Niu Q, Jiang L *et al.*Regulated intramembrane proteolysis of the AXL receptor kinase generates an intracellular domain that localizes in the nucleus of cancer cells. *The FASEB Journal*2016; 31: 1382–1397.
84.Tang K, Ling M. 89 Tie-2 regulates the stemness of prostate cancer cells. *European Journal of Cancer*2014; 50: 33.
85.Carpenter G, Liao H-J. Trafficking of receptor tyrosine kinases to the nucleus. *Experimental Cell Research*2009; 315: 1556–1566.
86.Pasquale EB. Eph receptors and ephrins in cancer: bidirectional signalling and beyond. *Nature Reviews Cancer*2010; 10: 165–180.
87.Husa A-M, Magić Ž, Larsson M, Fornander T, Pérez-Tenorio G. EPH/ephrin profile and EPHB2 expression predicts patient survival in breast cancer. *Oncotarget*2016; 7: 21362–21380.
88.Bagheri-Yarmand R, Sinha KM, Gururaj AE, Ahmed Z, Rizvi YQ, Huang S-C et al. A Novel Dual Kinase Function of the RET Proto-oncogene Negatively Regulates Activating Transcription Factor 4-mediated Apoptosis. Journal of Biological Chemistry 2015; 290: 11749–11761.
89.Chang H, Sung JH, Moon SU, Kim H-S, Kim JW, Lee JS. EGF Induced RET Inhibitor Resistance inCCDC6-RETLung Cancer Cells. *Yonsei Medical Journal*2017; 58: 9.
90.Green J, Nusse R, Amerongen RV. The Role of Ryk and Ror Receptor Tyrosine Kinases in Wnt Signal Transduction. *Cold Spring Harbor Perspectives in Biology*2013; 6. doi:10.1101/cshperspect.a009175.
